# Acute Respiratory Diseases among Troops with Especial Reference to Empyema

**Published:** 1919

**Authors:** 


**Acute Respiratory Diseases among Troops with Especial Reference to Empyema.** By L. S. Beals, B. F. Zimmerman and S. B. Marlow. *The Journal of Infectious Diseases*, November 1918.

Respiratory infections in 1917 and 1918 occurred among troops in waves, the greatest severity of the diseases being at the height of each wave. Not only were empyema and pneumonia more frequent and severe, but also tonsilitis, pharyngitis and bronchitis.

The most serious infections were characterized by septicemia with empyema, peritonitis, phlegmon and rarely arthritis, and also by pulmonary infection (pneumonia lobular or lobar in distribution) with consecutive empyema.

While patients who were passing through measles infections were more severely affected by the streptococcal invasion, the two infections should be regarded as separate, even though they were coincident. The empyema and pneumonia were not the result of the measles infection but of the prevailing severe respiratory infections of that period. The marked freedom from complications of the group of measles treated during the fall and winter of 1917, when general streptococcal infections were absent in camp, and the severe wave of pneumonia and streptococcal empyema of April 1918, when measles was on the wane, and which involved for the most part soldiers who had not had measles, are points in support of this view.

In a group of 115 consecutive cases of pneumonia and empyema, a definite history of an acute antecedent bronchitis, pharyngitis or tonsilitis was obtained in over 80 o/o of the cases.

In the majority of cases of empyema, a streptococcus has been the causative organism. When grown on blood agar it has been shown to be usually hemolytic. During the period of prevalence of empyema, streptococci were demonstrable in the majority of the throats of healthy soldiers as well as of patients entering the hospital with respiratory infections.

The clinical manifestations of empyema include rapid and profound toxemia, the quick formation in many cases of large amounts of pleural exudate, and a marked tendency to pocket formation by old and new adhesions in atypical locations.

The mortality was highest in the cases of empyema following measles, and the lowest in cases in which empyema was the primary condition.

At necropsy the outstanding features have been the finding of widespread lesions with a tendency to involve serous membranes, the occurrence of a severe bronchitis in the majority of cases, and a type of broncho-pneumonia so distributed as often to closely resemble a lobar process.

Early recognition of empyema is important, especially in the acute fulminant type of cases. The diagnosis depends on close attention to clinical signs. Dulness, the sense of resistance on percussion, and the diminution or absence of tactile fremitus are the most reliable signs.

The relative incidence of empyema complicating pneumonia was high : 50 o/o in one month.

Of 830 cases of measles and rubella, 44 o/o developed pneumonia and empyema; of this group empyema was the primary condition clinically in 70 o/o of the cases.

The control of such epidemics would appear to depend on the sanitation in barracks as regards ventilation; on adequate space and proper separation, especially in those even slightly affected with respiratory disease; on line officers not allowing overheated men to stand inactive in cold winds; on not indiscriminately giving triple typhoid inoculations at the very entrance of the soldier to camp without any regard to the respiratory infections he might have at the time; on careful attention to overexertion and exposure of soldiers having mild respiratory diseases; and on close attention to the details of equipment to ensure adequate clothing for the unacclimated raw recruit on his arrival.

